# Genome-wide analysis of the *SOS2* gene family in melon (*Cucumis melo* L.) and functional characterization of *MELO3C010334* in response to salt stress

**DOI:** 10.1186/s12870-026-08456-3

**Published:** 2026-02-27

**Authors:** Mengli Yang, Shen Liang, Changqing Xuan, Yufan Ma, Kehan Yang, Fei Chen, Man Zhang, Mengyun Hou, Kai Zhao

**Affiliations:** 1 Institute of Horticultural Research, Henan Academy of Agricultural Sciences, Zhengzhou, 450002 China; 2 College of Life Science and Technology, North Henan Medical University, Xinxiang, 453003 China; 3 College of Horticulture, Henan Agricultural University, Zhengzhou, 450046 China; 4 College of Agronomy, Henan Agricultural University, Zhengzhou, 450046 China

**Keywords:** Melon, *SOS2* gene, Salt stress, Gene expression

## Abstract

**Background:**

Soil salinity is a major abiotic stress that severely restricts the growth, yield, and quality of melon (*Cucumis melo* L.). The Salt Overly Sensitive (SOS) signaling pathway plays a crucial role in plant salt tolerance, in which *SOS2* acts as a central kinase regulating ion homeostasis and stress responses. However, the *SOS2* gene family in melon remains unclear.

**Results:**

Here, we identified 20 *SOS2* genes (*CmSOS2*) in the melon genome, distributed unevenly across nine chromosomes. All CmSOS2 proteins contain conserved pkinase and NAF domains, essential for kinase activity and calcium sensor binding. Phylogenetic analysis classified the genes into seven clades, indicating evolutionary conservation across species. Promoter analysis revealed abundant *cis*-elements associated with light, hormone, and stress responses. Expression profiling under salt stress and hormone treatments showed significant upregulation of *MELO3C010334* and *MELO3C007208*, suggesting their pivotal roles in stress signaling. Further functional validation confirmed that overexpression of *MELO3C010334* significantly enhanced salt tolerance in melon, promoted root growth, and improved Na^+^/K^+^ homeostasis in roots.

**Conclusions:**

This study represents the first systematic characterization of the genomic features and expression patterns of the SOS2 gene family in melon, and clarifies the positive regulatory role of *MELO3C010334* under salt stress. It provides important genetic resources and a theoretical basis for further understanding the molecular mechanisms of salt tolerance and for genetic improvement of salt tolerance in melon.

**Supplementary Information:**

The online version contains supplementary material available at 10.1186/s12870-026-08456-3.

## Background

Melon (*Cucumis melo* L.) belongs to the Cucurbitaceae family and is an important economically valuable fruit and vegetable crop widely cultivated for its rich content of various vitamins and minerals [1, 2]. However, with the large-scale adoption of protected cultivation techniques, long-term high-density planting and irrational irrigation practices have led to continuous accumulation of soil salinity. Salt stress causes chlorosis in melon leaves, reduces chlorophyll content, induces stomatal closure, and decreases the net photosynthetic rate, thereby hindering water uptake [3, 4]. The accumulation of excessive Na^+^ and Cl^−^ ions disrupts intracellular ion balance and inhibits the activity of key metabolic enzymes, further impeding the synthesis and accumulation of photosynthetic products and ultimately affecting melon growth and fruit development [5–7]. Therefore, a deep understanding of the molecular mechanisms underlying melon responses to salt stress, especially the identification of salt tolerance-related signaling pathways and key genes, is of great significance for enhancing melon salt tolerance, ensuring stable production, and expanding cultivation in saline-alkali lands.

Under high salinity conditions, when the Na^+^ concentration in the cytoplasm rises to toxic levels, plants activate the Na^+^/H^+^ antiporter system to exclude Na^+^ from the cell or sequester it into vacuoles, thereby maintaining a low cytosolic Na^+^ level [8–10]. This process primarily relies on the coordinated action of the Salt Overly Sensitive (SOS) protein located on the plasma membrane and the NHX family proteins located on the vacuolar membrane [11]. Its regulatory hub depends on the highly conserved SOS signaling pathway. Within the signaling network of plant responses to salt stress, the SOS pathway is currently the most clearly studied and functionally well-defined ion homeostasis regulation system [12–14]. This pathway centers on *SOS1* (a plasma membrane Na^+^/H^+^ antiporter), *SOS2* (a serine/threonine protein kinase), and *SOS3* (a calcium sensor protein), and is finely regulated by auxiliary factors such as *SOS4* and *SOS5*, working together to maintain cellular Na^+^/K^+^ homeostasis [15, 16]. SOS2, as the key central kinase in this pathway, possesses the dual function of perceiving Ca^2+^ signals and transducing them downstream [17, 18]. Its C-terminal contains a FISL motif, which allows it to bind to SOS3 upon Ca^2+^ signal activation, forming a *SOS3*-*SOS2* complex, this complex then phosphorylates and activates *SOS1*, promoting Na^+^ efflux [19]. Additionally, *SOS2* enhances the activity of the vacuolar membrane H^+^-ATPase and NHX-type Na^+^/H^+^ antiporters, facilitating the sequestration of Na^+^ into vacuoles [20, 21]. Beyond regulating ion homeostasis, *SOS2* can also activate antioxidant factors like NDPK2, CAT2, and CAT3 to enhance reactive oxygen species (ROS) scavenging capacity, thereby mitigating oxidative damage caused by salt stress [22].

Recent studies have shown that *Arabidopsis thaliana PKS5* negatively regulates the SOS signaling pathway [23]. The transcriptional repressor SSN1 regulates the degradation of the *SOS2*-*PIF4* complex through liquid-liquid phase separation, forming “salt bodies” [24]. The ESCRT-Ⅲ component *FYVE4* positively regulates salt tolerance by enhancing the *SOS1*-*SOS2* interaction [25]. *SOS2* can also maintain ammonium uptake by activating AMT1;1 to optimize the salt stress response [26]. In crops, tomato (*Solanum lycopersicum*) *SlSOS2* enhances salt tolerance by regulating root Na^+^ efflux, xylem Na^+^ loading, and vacuolar sequestration [27, 28]. Furthermore, *SOS* homologous genes have been identified in species such as rice (*Oryza sativa* L.), wheat (*Triticum aestivum* L.), and sunflower (*Helianthus annuus* L.), indicating the functional conservation of this pathway within the plant kingdom [29–31]. However, compared to model plants and major food crops, the genomic characteristics, member evolution, and expression patterns of the SOS2 family in cucurbit crops, particularly melon, remain poorly studied.

Based on the genome-wide sequence of melon, this study conducted a systematic identification and analysis of the SOS2 family. We first identified family members using sequence homology alignment and conserved domain recognition. We analyzed their physicochemical properties, conserved motifs, gene structures, and chromosomal distribution. Phylogenetic trees were constructed to investigate evolutionary relationships and homology with other species. Potential regulatory patterns were analyzed through promoter element prediction, and gene expansion and evolutionary drivers were revealed using collinearity analysis. Quantitative real-time PCR (qRT-PCR) was employed to detect their expression changes under salt stress and hormone treatments, elucidating the specific expression characteristics of the SOS2 family. Candidate genes closely associated with salt tolerance in melon were screened and further functionally validated through a hairy‑root transformation system. Overexpression assays, combined with phenotypic observation, root architecture analysis, and ion content measurement, demonstrated the critical role of key genes in regulating ion homeostasis and enhancing salt tolerance. This study provides key genetic resources for elucidating the molecular mechanisms of salt tolerance in melon and lays a theoretical and material foundation for subsequent molecular breeding.

## Materials and methods

### Plant materials and experimental treatments

The experiment utilized the thick-skinned melon cultivar ‘Yuyuan 28’, developed by the Horticulture Research Institute of Henan Academy of Agricultural Sciences. Plump and uniform seeds were selected, subjected to soaking and germination treatments, and sown in trays upon radicle emergence. At the one-leaf-one-heart stage, seedlings were transplanted into 6 cm × 6 cm nutrient pots filled with a sterile peat-based substrate mixture and cultivated in a light incubator under the following conditions: light intensity 30,000 lx, photoperiod 14-h light/10-h dark, temperature 28 °C/18°C, and relative humidity 60%. When the seedlings reached the three-leaf stage, they were subjected to the following abiotic stress treatments: To apply salt stress, plants were irrigated with NaCl solutions at concentrations of 100, 200, and 300 mM prepared in deionized water [32]. The solution was applied as a drench until it leached from the bottom of the pots, ensuring uniform and acute stress to the entire root zone. For hormone treatments, solutions of 1 mM salicylic acid (SA), 100 µM abscisic acid (ABA), and 100 µM methyl jasmonate (MeJA). Root and third true leaf samples were collected at 0, 12, 24, and 48 h after treatment, rapidly frozen in liquid nitrogen, and stored at -80 °C for subsequent RNA extraction.

### Identification of the *CmSOS2* gene family and analysis of protein physicochemical properties

The whole-genome sequence, gene annotation file, and protein sequence of melon were obtained from the CuGenDB (http://cucurbitgenomics.org/). The hidden Markov model (HMM) profile of the NAF domain (PF03822) was downloaded from the Pfam database (http://pfam.xfam.org). Candidate proteins containing the SOS2 domain were screened using local HMMER software with an *E*-value threshold of 1e^− 15^, resulting in the initial identification of 20 *SOS2* genes. conserved domains were further verified via the SMART website (https://smart.embl.de/). The number of amino acids, molecular weight, and theoretical isoelectric point of the proteins were analyzed using ExPASy ProtParam (https://web.expasy.org/protparam/). Subcellular localization was predicted with Cell-PLoc 2.0 (http://www.csbio.sjtu.edu.cn/bioinf/Cell-PLoc-2/). The chromosomal locations of *CmSOS2* genes were determined based on the melon genome database and visualized using MG2C (https://qiaoyundeng.github.io/). Gene structures were predicted using the SoftBerry platform (http://www.softberry.com/).

### Phylogenetic and protein sequence alignment analysis of *SOS2* genes in different plants

Protein sequences annotated as SOS2 were downloaded from the *Arabidopsis* (https://www.arabidopsis.org/), cucumber (*Cucumis sativus* L.), watermelon (*Citrullus lanatus*), and melon (http://cucurbitgenomics.org/) genome databases. Multiple sequence alignment was performed using MUSCLE in MEGA11, and a phylogenetic tree was constructed using the neighbor-joining (NJ) method [33]. The tree was visualized and annotated using the iTOL online tool (https://itol.embl.de/). Conserved motifs in the SOS2 protein sequences were identified using the MEME suite (https://meme-suite.org/meme/) with the number of motifs set to 15. Protein secondary structure alignment was conducted using ESPript 3.0 (https://espript.ibcp.fr/). The three-dimensional structure of the SOS2 protein was obtained from the AlphaFold database (https://alphafold.ebi.ac.uk/) in PDB format and visualized using PyMOL software.

### Analysis of synteny, promoter *cis*-acting elements, and protein interaction networks

Synteny analysis among *Arabidopsis*, cucumber, watermelon, and melon was performed using the JCVI package to elucidate the evolutionary relationships of the *SOS2* gene family [34]. Putative *cis*-acting elements within the 2000 bp promoter region upstream of the *CmSOS2* genes were predicted using the PlantCare database (http://bioinformatics.psb.ugent.be/webtools/plantcare/html/), and a heatmap of element distribution was generated using TBtools-II [35]. The protein-protein interaction network of CmSOS2 protein was predicted using the STRING database (https://cn.string-db.org/).

### Expression analysis of *CmSOS2* in different tissues

Expression data of *CmSOS2* genes in various tissues and developmental stages (including root, stem, leaf, flower, and fruit) of melon were obtained from the Melonet DB database (https://melonet-db.dna.affrc.go.jp/ap/top) and used to generate a tissue-specific expression heatmap [36, 37].

### qRT-PCR analysis of *CmSOS2* under abiotic stresses

Root tissues from different treatments were ground in liquid nitrogen, and total RNA was extracted using a plant total RNA extraction kit (Vazyme Biotech Co., Ltd, China). RNA integrity was verified by agarose gel electrophoresis. cDNA was synthesized from 1 µg of total RNA using a reverse transcription kit (Vazyme). Primers were designed with Primer 5.0, with amplicon lengths between 150 and 300 bp (Table S1). *CmActin7* was used as the internal reference gene. qRT-PCR was performed using SYBR qPCR Master Mix (Vazyme) in a 20 µL reaction system containing 10 µL of Master Mix, 0.5 µL each of forward and reverse primers, 2 µL of cDNA, and 7 µL of ddH₂O. The reaction protocol consisted of: pre-denaturation at 95 °C for 30 s; 40 cycles of 95 °C for 10 s and 60 °C for 30 s; and a melt curve stage of 95 °C for 15 s, 60 °C for 60 s, and 95 °C for 15 s. Relative gene expression levels were calculated using the 2^–ΔΔCt^ method. Three biological replicates were performed, each with three technical replicates. Data analysis and graph preparation were conducted using GraphPad Prism 8.0.

### Hairy Root Transformation in Melon

The constructed target fusion protein expression vector was introduced into Agrobacterium tumefaciens strain K599 competent cells using the freeze-thaw method. The hairy-root transformation system used in this study has been successfully applied for functional analysis in melon [38]. Transformants were plated on LB solid medium containing appropriate antibiotics and incubated inverted at 28 °C for 36 h. Single colonies were picked, expanded in liquid culture, and bacterial cells were harvested. Then, 1 µL of 0.5 M acetosyringone was added to the bacterial suspension, followed by vortexing for 30 s. Uniform 5-day-old melon seedlings (salt-sensitive cultivar ‘Yulu’) were selected, and the primary roots were aseptically excised on a sterile bench. The cut ends were immediately dipped into an Agrobacterium suspension with an OD_600_ of 0.8. The inoculated seedlings were carefully transplanted into sterilized vermiculite substrate and co-cultivated in darkness at 30 °C for 2 days, during which the substrate was kept moist to maintain bacterial activity. After co-cultivation, the seedlings were transferred to half-strength Hoagland’s nutrient solution for hydroponic culture.

## Results

### Identification and physicochemical characterization of the *CmSOS2* family

In this study, we conducted a genome-wide systematic identification of the *SOS2* gene family in melon, revealing a total of 20 members. Comprehensive analysis of their physicochemical properties was performed (Table S2). The results indicated that the amino acid lengths of these proteins range from 365 to 497, with molecular weights (Mw) varying between 41.02 and 56.11 kDa, demonstrating considerable variability. The theoretical isoelectric point (pI) spans from 5.60 to 9.25, suggesting that these proteins may exhibit distinct charge states under different pH conditions. The instability index (Ii) values ranged from 28.96 to 48.85. Among them, *MELO3C026055* (Ii = 28.96) was predicted to be highly stable, whereas *MELO3C010334* (Ii = 48.85) was classified as unstable. The aliphatic index was generally high (77.29–93.99), indicating that the SOS2 protein family possesses good thermal stability. All members exhibited negative Grand Average of Hydropathicity (GRAVY) values, ranging from − 0.54 to -0.16, confirming their hydrophilic nature and suggesting potential involvement in cytoplasmic and membrane-associated signal transduction processes.

Subcellular localization predictions revealed that SOS2 proteins are widely distributed across multiple compartments, including the cytoplasm (*MELO3C002661*, *MELO3C011108*), chloroplast (*MELO3C002766*, *MELO3C007208*), nucleus (*MELO3C005987*,* MELO3C006758*), endoplasmic reticulum (*MELO3C010234*), plasma membrane (*MELO3C006483*), and cytoskeleton (*MELO3C026055*). This diverse subcellular distribution implies that the *SOS2* gene family may participate in regulating various biological processes such as environmental sensing, ion homeostasis, and stress responses. Taken together, the *SOS2* gene family in melon exhibits significant diversity in Mw, pI, and subcellular localization.

### Chromosomal localization and gene structure analysis of the *CmSOS2* gene family

**Fig. 1:**
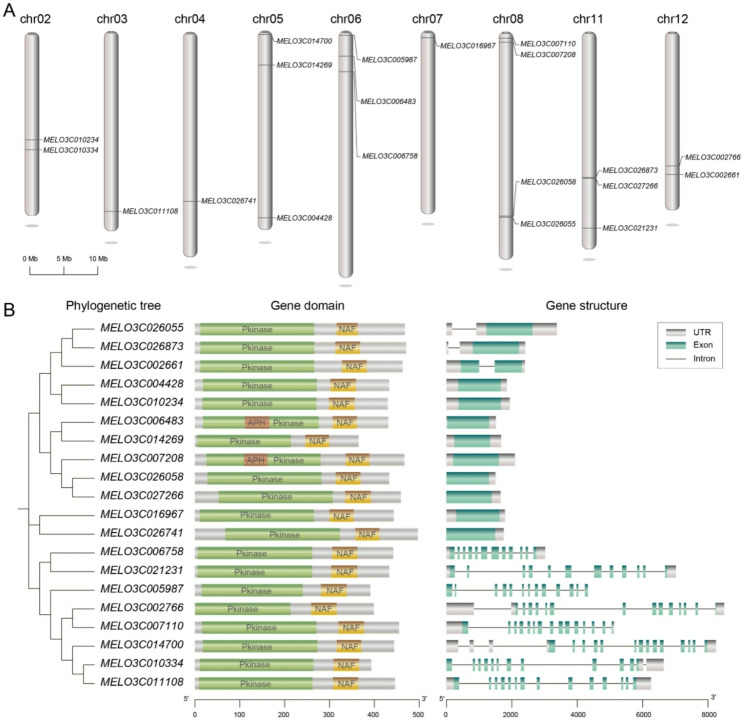
Genome-wide information related to the melon *SOS2* gene family, including its distribution on chromosomes, conserved domains, and gene structure. **A** Chromosomal localization of 20 *CmSOS2* members, scale bar 5 Mb. **B** Analysis of the phylogenetic tree, conserved domains, and gene structure of the melon *SOS2* gene family. The phylogenetic tree was constructed using the neighbor-joining (NJ) method. Green represents the pkinase domain, yellow represents the NAF domain, and red represents the APH domain; gray boxes indicate UTR regions, cyan boxes represent exons, and black lines represent introns

Chromosomal localization analysis revealed that these *SOS2* genes are unevenly distributed across nine chromosomes (Chr2, Chr3, Chr4, Chr5, Chr6, Chr7, Chr8, Chr11, and Chr12) (Fig. 1A). Notably, Chr8 contained the highest number of *SOS2* genes (4), while Chr2 and Chr3 each carried only 1–2 genes. No *SOS2* genes were detected on Chr1, Chr9, or Chr10, suggesting uneven evolutionary expansion or loss of this gene family across the melon genome. Phylogenetic analysis based on amino acid sequences using the NJ method classified the *CmSOS2* genes into four distinct clades (Fig. 1B). Conserved domain analysis indicated that all members contain a canonical pkinase domain, which is essential for kinase activity and participation in phosphorylation cascades. Most members also possess an NAF domain, known to mediate interaction with SOS3-like calcium sensors and playing a critical role in abiotic stress responses such as salt tolerance. Additionally, several genes (*MELO3C014269* and *MELO3C007208*) contain an APH domain, implying potential functional specialization in protein interaction networks or specific stress adaptation.

Gene structure analysis revealed high conservation of exon-intron organization among members within the same phylogenetic clade (Fig. 1B). For instance, genes in Clade I (*MELO3C026055* and *MELO3C026873*) exhibit similar exon numbers and lengths, suggesting conserved transcriptional and translational regulation. In contrast, notable structural divergence was observed between clades. *MELO3C021231* and *MELO3C005987* contain more exons and complex intron configurations, which may reflect functional diversification related to tissue-specific expression or nuanced stress response regulation.

### Phylogenetic classification and motif comparative analysis of *SOS2* gene family members across species

**Fig. 2:**
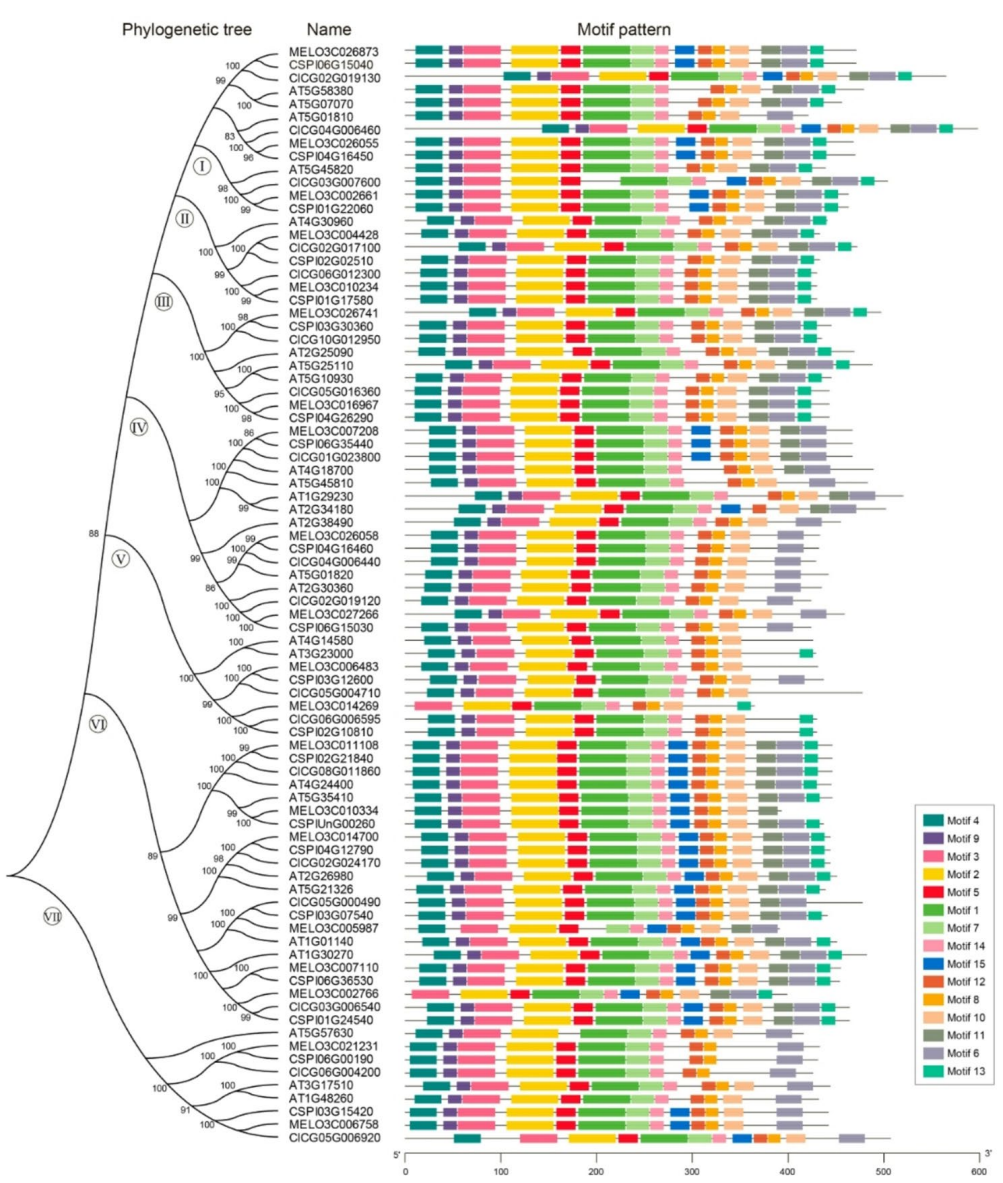
Phylogenetic relationships and conserved motif composition analysis of SOS2 amino acid sequences from multiple species. The left panel presents a phylogenetic tree constructed using the NJ method with 1,000 bootstrap replicates. The analysis included SOS2 amino acid sequences from melon, *Arabidopsis*, cucumber, and watermelon accessions. The phylogenetic tree is divided into seven distinct clades (I to VII). The right panel displays the distribution patterns of conserved motifs (Motifs 1 to 15) predicted by the MEME suite. The color code in the legend corresponds to each specific motif. This schematic intuitively represents the presence, sequential order, and relative positions of these conserved motifs within each gene

To investigate the evolutionary relationships and conserved functional domains of the *SOS2* gene family, we performed a comparative analysis using protein sequences from melon, *Arabidopsis*, cucumber, and watermelon (Table S3). A NJ phylogenetic tree was constructed based on amino acid sequences, and conserved motifs were identified using the MEME suite (Fig. 2). The phylogenetic analysis clearly classified all *SOS2* genes into seven distinct clades (I-VII). Notably, most clades contained *SOS2* homologs from multiple species. Specifically, Clade Ⅱ includes MELO3C004428 and MELO3C010234 from melon, AT4G30960 from *Arabidopsis*, and watermelon (CICG02G017100 and CICG06G012300), suggesting strong evolutionary conservation of core functions among these genes.

A total of 15 conserved motifs (motifs 1–15, represented by different colored boxes) were identified. Motif composition and distribution patterns revealed that all genes within Clade I contain core motifs such as motif 1, 2, 3, and 5, which are associated with the pkinase domain (involved in phosphorylation and regulation of downstream targets) and the NAF regulatory domain (medi interaction with SOS3-like calcium sensors). This implies functional conservation within this clade in processes such as ion homeostasis and salt stress response. Most SOS2 proteins contain more than 10 conserved motifs, which are predominantly clustered in the central region of the sequences. This distribution is consistent with the centralized organization of functional domains in SOS2 proteins and further supports the reliability of the motif analysis.

### Amino acid alignment and three-dimensional structure analysis of the *CmSOS2* gene family

**Fig. 3:**
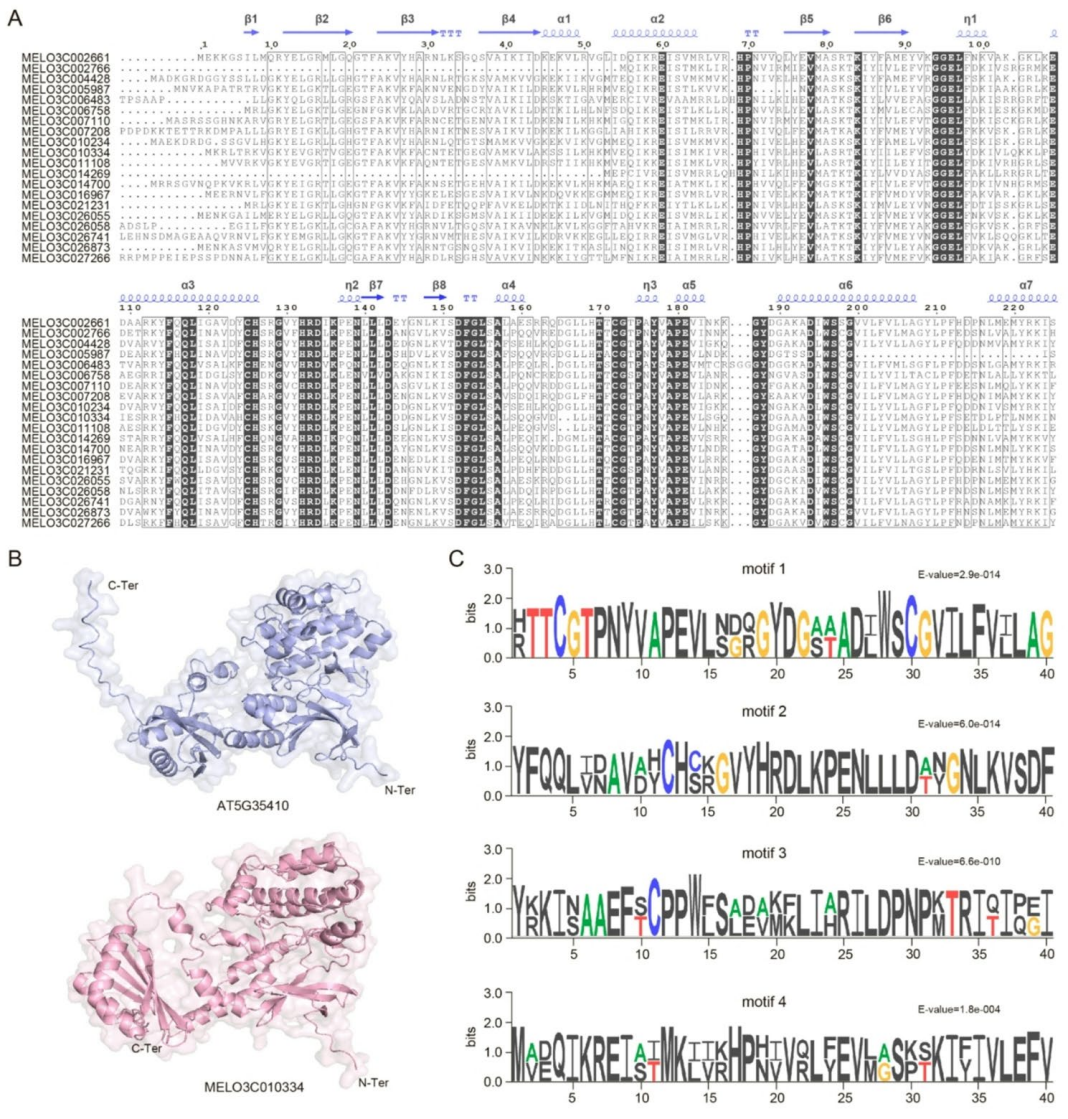
Analysis of sequence conservation, protein tertiary structure, and conserved motifs of the melon *SOS2* gene family. **A** Multiple sequence alignment of amino acid sequences of *CmSOS2* family members. The alignment includes amino acid sequences encoded by several CmSOS2 proteins. Black shaded regions indicate highly conserved residues, predicted secondary structure elements, β1 to β8 (β strands), α1 to α7 (α helices), and η1 to η3 (η helices), are annotated above the alignment, reflecting conserved sequence and structural motifs among family members. **B** Tertiary structure modeling of SOS2 proteins. Simulated structures of the *Arabidopsis* reference protein AT5G35410 (blue) and the melon CmSOS2 protein MELO3C010334 (pink) are shown, with N-terminus (N-ter) and C -terminus (C-ter) ends labeled. The similarity in their spatial folding patterns illustrates the evolutionary structural conservation of SOS2 proteins. **C** Sequence logo analysis of conserved motifs in the *CmSOS2* family. Sequence features of four core conserved motifs are displayed. The height of each amino acid residue represents its conservation level at that position, with colors differentiating residue types. The *E*-value on the right indicates the statistical significance of each motif, highlighting their core sequence attributes and functional importance within the family

To investigate the sequence conservation of the melon *SOS2* gene family, a multiple sequence alignment was performed on the identified SOS2 protein amino acid sequences (Fig. 3A). The results revealed a large number of highly conserved amino acid residues among different SOS2 members (black shaded regions), with particularly high consistency observed within secondary structural elements such as α-helices (α1-α7), β-sheets (β1-β8), and η-helices (η1-η3). In *Arabidopsis*, SOS2 (AT5G35410) is known to interact with the calcium sensor protein SOS3 under salt stress, forming a complex that phosphorylates and activates the plasma membrane Na^+^/H^+^ antiporter SOS1, thereby driving sodium efflux to maintain cellular ion homeostasis [39]. To further analyze the structural basis of this function, the known functional homolog AT5G35410 from *Arabidopsis* was selected as a reference for homology-based three-dimensional structure modeling with melon MELO3C010334 (Fig. 3B). The results indicated that the two proteins exhibit high spatial conformational similarity, both adopting the typical protein kinase domain fold, including an N-terminus (N-ter) catalytic core and a C-terminus (C-ter) regulatory region. This structural similarity further supports the potential functional conservation between melon SOS2 and *Arabidopsis* SOS2. Additionally, conserved motifs within the SOS2 family were predicted using MEME and further analyzed with Sequence logo (Fig. 3C). Four core conserved motifs (motifs 1–4) were identified. Among these, motif 1 contains conserved residues such as “HTTCGTPNVY”, which correspond to the ATP-binding and catalytic phosphorylation region within the pkinase domain. Based on these findings, we speculate that the melon *SOS2* gene may play an important biological role in the response to salt stress.

### Analysis of synteny, promoter *cis*-elements, and protein interaction network of the melon *SOS2* gene family

**Fig. 4:**
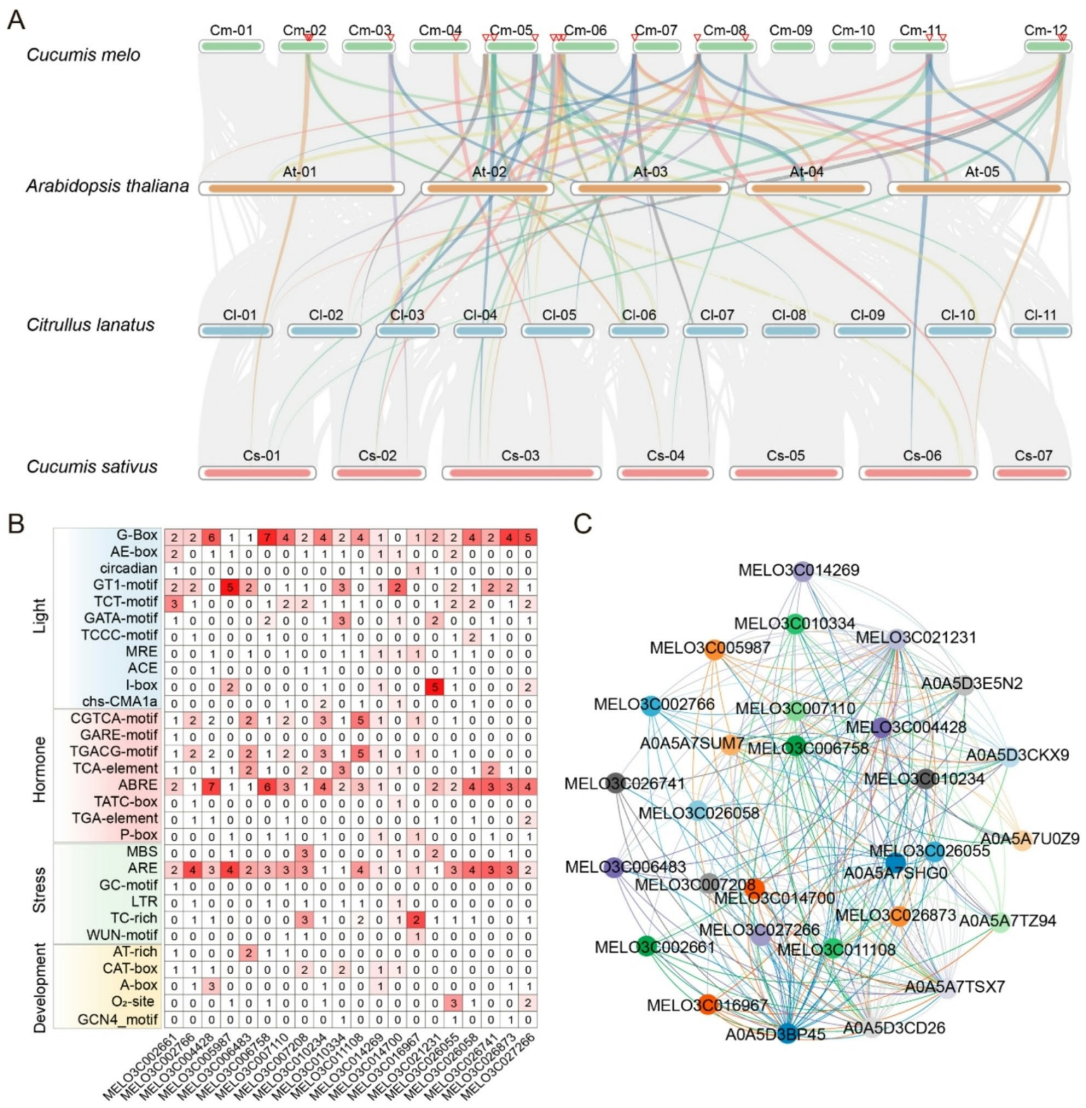
Analysis of interspecific collinearity, promoter *cis*-acting elements, and protein interaction network of *SOS2* gene family in melon. **A** Collinearity analysis of *SOS2* genes among melon, *Arabidopsis*, watermelon, and cucumber. Chromosomes of melon (Cm01 to Cm12), *Arabidopsis thaliana* (At01 to At05), *Citrullus lanatus* (Cl01 to Cl11), and *Cucumis sativus* (Cs01 to Cs07) are displayed. Colored lines connect collinear *SOS2* homologous gene pairs across species, illustrating evolutionary conservation and genomic synteny of the gene family. **B** Distribution of *cis*-acting elements in the 2000 bp promoter region upstream of the melon *SOS2* genes. Rows represent functional categories of *cis*-elements. Light response (light), hormone response (e.g., ABA, GA), stress response (e.g., drought, low temperature), and developmental regulation (e.g., C-box, AE-box, GT1-motif, TCC-motif). Columns correspond to individual *CmSOS2* genes. Cell color intensity and values indicate the abundance of each element type in the promoter region. **C** Predicted protein-protein interaction network of CmSOS2 proteins, generated using the STRING database (version 12.0). Nodes with the MELO3C prefix represent melon SOS2 family members, while A0A-prefixed nodes denote putatively interacting proteins predicted by the database. Edges indicate predicted functional associations

To investigate the evolutionary conservation of the melon *SOS2* gene family, a systematic synteny analysis was conducted among melon, *Arabidopsis*, watermelon, and cucumber (Fig. 4A). The synteny analysis indicates that *SOS2* genes are located within extensive conserved genomic blocks across melon, *Arabidopsis*, watermelon, and cucumber, supporting the conservation of their core functions in plant salt stress responses. The calculated Ka/Ks ratios are all significantly less than 1, indicating that *SOS2* genes have undergone strong purifying selection during evolution, and their functions are highly conserved (Fig S1; Table S4). Although homology is higher within the Cucurbitaceae family (melon, watermelon, cucumber), none of the Ka/Ks values from all comparisons show signs of significant positive selection. This suggests that the functional stability of this gene family has been maintained without large-scale adaptive divergence.

Analysis of *cis*-acting elements in the promoter regions (2,000 bp upstream of the transcription start site) of melon *SOS2* genes identified various functional elements (Fig. 4B), including light-responsive elements (G-Box, AE-box), hormone-responsive elements (ABRE for ABA, TGA-element for SA, and GARE-motif for GA), stress-responsive elements (MBS and LTR), and development-related elements (CAT-box and A-box, ). The composition of *cis*-elements varied significantly among different *SOS2* genes. For instance, *MELO3C004428* was highly enriched in ABRE elements, implying its potential role in ABA-mediated response. In contrast, *MELO3C002661* and *MELO3C006758* contained numerous light-responsive elements, suggesting its involvement in photoperiod or photosynthesis-related growth regulation. This diversity in *cis*-element profiles provides a transcriptional regulatory basis for the functional divergence of *SOS2* genes in various biological processes, including growth development and stress response. Protein-protein interaction prediction (Fig. 4C) further revealed potential interaction networks involving melon SOS2 proteins. In the constructed network, core SOS2 proteins such as MELO3C014269, MELO3C010334, and MELO3C021231 served as hub nodes. The functional diversity of promoter *cis*-elements and the complexity of the protein interaction network further support its potential involvement in multifunctional regulation, including abiotic stress response and growth development.

### Expression profiling of the *SOS2* gene family in melon

**Fig. 5:**
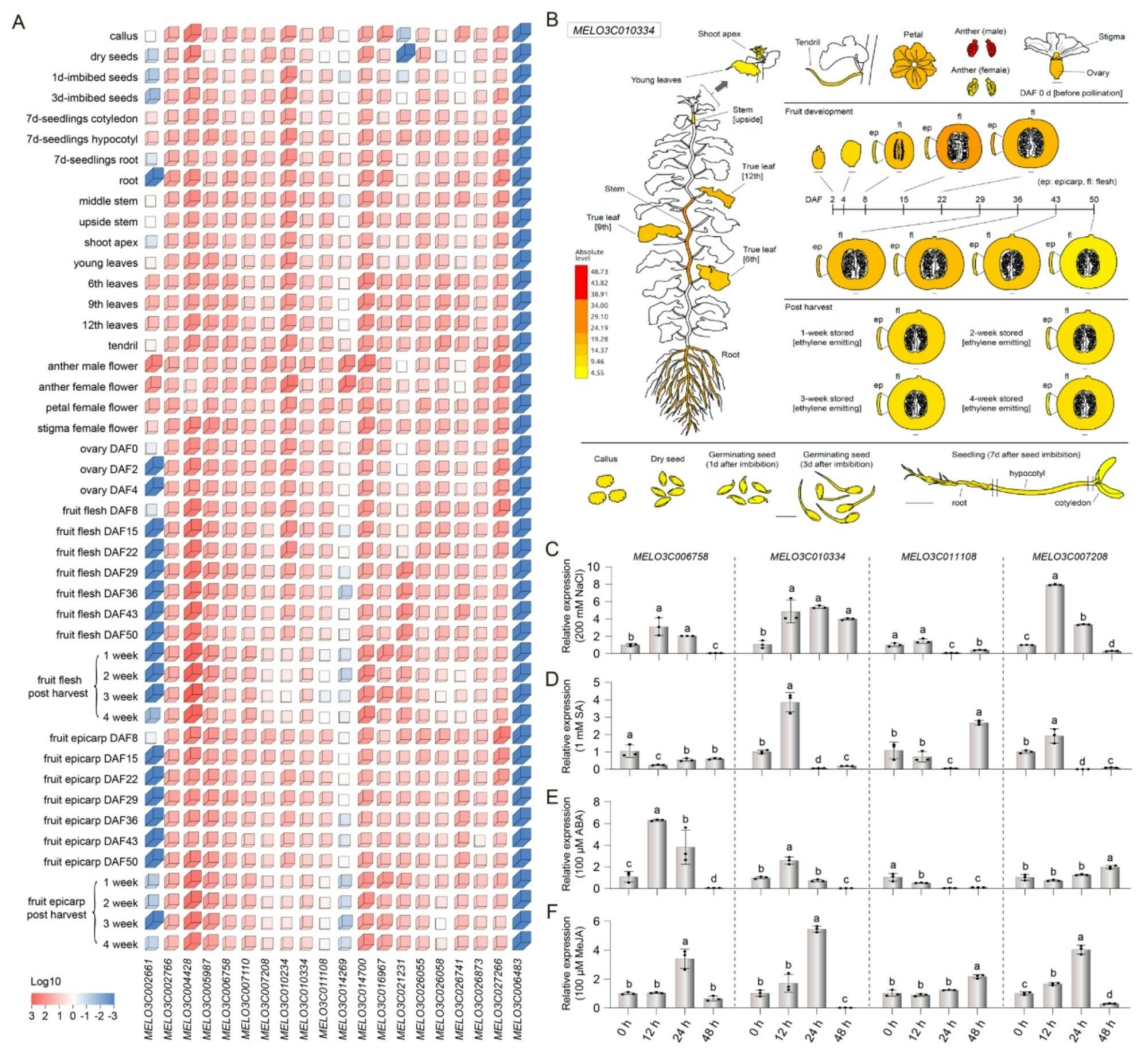
Expression pattern analysis of *SOS2* gene family in melon. **A** Expression heatmap of *SOS2* gene family members in melon across various tissues and developmental stages, including callus, seeds at different days after germination, various parts of seedlings, stems, leaves, flowers, ovaries, fruits at different days after flowering (DAF), and postharvest storage periods. Color gradient from blue to red represents the log10-transformed gene expression values, illustrating the expression specificity of family members in different tissues and developmental stages. **B** Schematic representation of the expression levels of the melon gene *MELO3C010334* across different tissues and developmental processes. Quantitative expression analysis of representative *SOS2* genes in melon (from Melonet DB [36, 37]). The relative expression levels of *MELO3C006758*, *MELO3C010334*, *MELO3C021108*, and *MELO3C007208* at different time points (0, 12, 24, and 48 h) after salt stress treatment (**C**), salicylic acid (**D**), abscisic acid (**E**), and methyl jasmonate (**F**). Different lowercase letters above the bars indicate significant differences (*P* < 0.05) as determined by one-way ANOVA followed by Duncan’s multiple comparison test

To elucidate the expression patterns of the *SOS2* gene family in melon, we generated a heatmap based on publicly available transcriptome data [36, 37], depicting their expression across various tissues and developmental stages (Fig. 5A; Table S5). In vegetative organs, *MELO3C010334* and *MELO3C021231* showed the highest expression levels (FPKM > 15) in roots and hypocotyls, suggesting their potential roles in ion uptake and transport (Fig. 5B). *MELO3C007208* was specifically highly expressed in leaves, possibly associated with calcium signaling in chloroplasts.

In reproductive organs, *MELO3C006758* was significantly upregulated during early ovary development 0 day after flowering (DAF), while *MELO3C026055* exhibited sustained high expression during fruit ripening (29–50 DAF), implying functional specialization of this family in floral organ differentiation and fruit quality formation. Developmental dynamic analysis revealed the most pronounced expression shift from seed germination to the seedling stage, with 11 genes upregulated more than 2-fold. Among these, *MELO3C014269* showed the highest induction (4.3-fold). These spatiotemporal expression patterns are consistent with the distribution of tissue-specific *cis*-elements (CAT-box, A-box) in the promoters, reflecting the decisive role of transcriptional regulation in functional partitioning.

### qRT-PCR analysis of *CmSOS2* genes under abiotic stresses

To investigate the response of CmSOS2 family members to NaCl stress, a concentration gradient of NaCl (100, 200, and 300 mM) was applied. The results indicated that treatment with 200 mM NaCl elicited the most pronounced changes in the expression of several *CmSOS2* genes (Fig. S2). To investigate the abiotic stress response of the CmSOS2 family, four representative genes (*MELO3C006758*, *MELO3C010334*, *MELO3C011108*, and *MELO3C007208*) were selected for in-depth analysis based on their phylogenetic distribution across different clades, promoter *cis*-element profiles, and preliminary stress-responsive expression patterns. The expression dynamics of these selected genes were then analyzed under NaCl, SA, ABA, and MeJA treatments at 0, 12, 24, and 48 h post‑treatment using qRT-PCR. Salt stress rapidly induced the expression of several *SOS2* genes (Fig. 5C). *MELO3C010334* showed the most significant response, with a 3.0-fold increase at 12 h (*P* < 0.01), peaking at 24 h (4.0-fold). *MELO3C006758* was upregulated 2.0-fold at 12 h but decreased markedly by 24 h. *MELO3C007208* increased sharply by 6.0-fold at 12 h. In contrast, *MELO3C011108* showed no significant change throughout the treatment (*P* > 0.05), indicating functional divergence among *SOS2* genes in salt stress response.

Under SA treatment (Fig. 5D), *MELO3C010334* was significantly upregulated 2.0-fold at 12 h (*P* < 0.05) but decreased rapidly by 24 h, displaying a quick activation and attenuation pattern. *MELO3C007208* was also upregulated 0.9-fold at 12 h (*P* < 0.05), suggesting its potential involvement in SA signaling. ABA treatment sustainably activated some *SOS2* genes (Fig. 5E). *MELO3C006758* reached a peak expression of 5.0-fold at 12 h (*P* < 0.01) and remained at a high level (2.0-fold) at 24 h. *MELO3C010334* was upregulated 1.5-fold at 12 h (*P* < 0.05), indicating their likely roles in ABA signal transduction. In response to MeJA (Fig. 5F), both *MELO3C010334* and *MELO3C007208* showed a delayed response, peaking at 24 h with upregulation of 4.0-fold and 3.0-fold (*P* < 0.01), respectively, implying their participation in JA-mediated stress responses. Taken together, *MELO3C010334* and *MELO3C007208* exhibited significant responses to multiple treatments, including salt, SA, ABA, and MeJA, with notably strong upregulation under salt stress. This suggests that these two genes may play crucial roles in melon’s response to abiotic stresses.

### Overexpression of *MELO3C010334* significantly enhances salt tolerance in melon

**Fig. 6:**
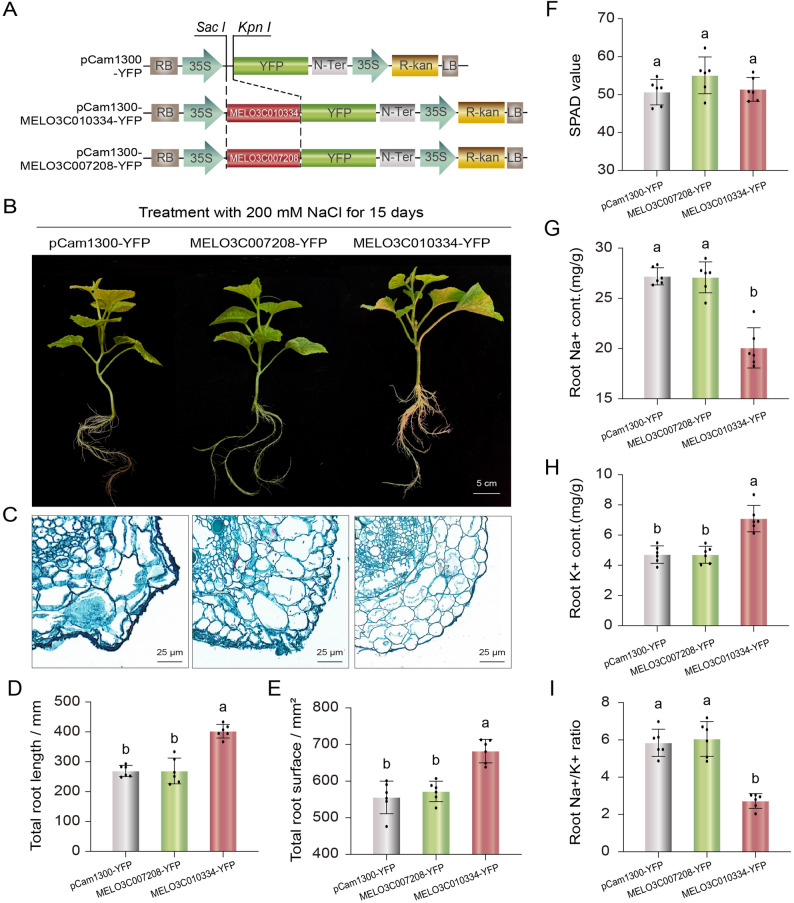
Functional analysis of *MELO3C007208* and *MELO3C010334* genes in melon under salt stress. **A** Schematic diagram of recombinant vector construction. *Sac I* and *Kpn I* are the key restriction sites used for construction. **B** Phenotype of melon plants after salt stress treatment: growth status of plants transformed with pCam1300-YFP (empty vector), MELO3C007208-YFP, and MELO3C010334-YFP after 15 days of treatment with 200 mM NaCl; scale bar = 5 cm. **C** Observation of root tissue cross sections. Root tissue sections of melon after salt stress treatment; scale bar = 25 μm. **D** Total root length after 15 days of salt stress. **E** Total root surface area after 15 days of salt stress. **F** SPAD value (relative chlorophyll content). **G** Measurement of Na^+^ content in roots. **H** Measurement of K^+^ content in roots. **I** Statistical analysis of Na^+^/K^+^ ratio in roots. Datas are presented as mean ± SD. ** indicates extremely significant difference compared with the empty vector group (*n* = 6, *P* < 0.01)

To elucidate the biological functions of *MELO3C007208* and *MELO3C010334* in melon response to salt stress, the coding sequences of these two genes were separately cloned into the pCambia1300-YFP vector via restriction enzyme digestion and ligation. This successfully constructed the recombinant overexpression vectors MELO3C007208-YFP and MELO3C010334-YFP (Fig. 6A; Fig. S3), followed by functional verification. After 15 days of treatment with 200 mM NaCl, phenotypic observations revealed that melon plants overexpressing MELO3C010334-YFP exhibited significantly better root growth compared to those harboring the empty vector or overexpressing MELO3C007208-YFP (Fig. 6B). Root tissue section analysis showed that under salt stress, root cells of plants carrying the empty vector or overexpressing MELO3C007208-YFP were disorganized, whereas root cells of plants overexpressing MELO3C010334-YFP remained structurally intact without obvious damage (Fig. 6C). Further quantitative analysis indicated that, after salt stress, total root length and total root surface area of plants overexpressing MELO3C010334-YFP were significantly higher than those of the empty vector control and plants overexpressing MELO3C007208-YFP (*P* < 0.01) (Fig. 6D, E). Measurement of relative chlorophyll content (SPAD values) showed no significant differences among the three groups (Fig. 6F). Compared to the empty vector, plants overexpressing MELO3C007208-YFP showed no significant change in K^+^ content, while Na⁺ content increased by approximately 3.3% on average, and the Na^+^/K^+^ ratio increased by approximately 3.4% on average. In contrast, plants overexpressing MELO3C010334-YFP exhibited a 33.7%-71.1% increase in K^+^ content, a 21.0%-37.2% decrease in Na⁺ content, and a significant 44.7%-62.9% reduction in the Na^+^/K^+^ ratio, demonstrating a clear trend toward improved ion homeostasis (Fig. 6G-I). Comprehensive analysis indicates that overexpression of *MELO3C010334* significantly improves root growth under salt stress, effectively regulates the balance of Na^+^ and K^+^ in roots, and thereby enhances salt tolerance in melon. These results directly confirm that *MELO3C010334* plays a key positive regulatory role in melon response to salt stress.

## Discussion

This study represents the first genome-wide systematic identification of SOS2 genes in melon. A comprehensive analysis of their physicochemical properties, gene structures, conserved motifs, and phylogenetic relationships revealed the potential important role of this family in the salt stress response of melon. The results indicate that melon SOS2 proteins exhibit significant differences in Mw, pI, and subcellular localization, suggesting possible functional divergence. Chromosomal distribution analysis showed an uneven distribution of *SOS2* genes in the melon genome, with the highest number on Chr8 (4 genes), while no family members were detected on Chr1, Chr9, or Chr10. This suggests that species-specific gene duplication or loss events may have occurred during evolution. Phylogenetic analysis divided the 20 members into four clades. Gene structures were highly conserved within the same clade but showed clear differences between clades. Conserved domain analysis confirmed that all members contain the canonical pkinase domain essential for kinase activity, and most possess the NAF domain, a key module for interacting with SOS3-like calcium sensors. This structural foundation is consistent with the core SOS pathway mechanism, where the Ca^2+^-activated SOS3-SOS2 complex phosphorylates and activates the SOS1 Na^+^/H^+^ antiporter to drive Na^+^ efflux, a mechanism conserved from *Arabidopsis* to tomato [40–42]. We hypothesize that core candidates like *MELO3C010334* may function similarly, potentially phosphorylating melon homologs of SOS1 or vacuolar NHX transporters to maintain ion homeostasis under salt stress. Specifically, the presence of the NAF domain in most CmSOS2 proteins implies their capacity to bind calcium sensors such as CmSOS3/SCaBP, forming a regulatory complex analogous to the *Arabidopsis* SOS3-SOS2 module [19]. This complex likely targets and phosphorylates CmSOS1 or vacuolar CmNHX transporters, thereby enhancing Na^+^ extrusion or sequestration-a mechanistic hypothesis that warrants future validation through protein interaction and kinase assays.

Cross-species comparison and collinearity analysis further revealed the evolutionary relationships between melon *SOS2* genes and their homologs in species such as *Arabidopsis*, cucumber, and watermelon. The phylogenetic tree showed that most clades contain members from multiple species. For example, Clade Ⅱ includes melon (MELO3C004428 and MELO3C010234), *Arabidopsis* (AT4G30960), and watermelon (CICG02G017100 and CICG06G012300), indicating strong evolutionary conservation in core functions. However, compared to the well-characterized SOS2 genes in *Arabidopsis* and tomato, which primarily mediate root Na^+^ efflux and vacuolar compartmentalization, several melon SOS2 members exhibit distinctive expression patterns that may reflect cucurbit-specific adaptations. For instance, in tomato, *SlSOS2* is strongly expressed in roots and contributes to radial Na^+^ transport and xylem loading, whereas in melon, *MELO3C007208* shows pronounced leaf-specific expression [41]. This suggests a potential role in protecting photosynthetic tissues-a critical adaptation for a fruit-bearing crop whose yield depends heavily on canopy performance under stress. Such leaf-preferential expression might implicate *MELO3C007208* in chloroplast-localized calcium signaling or in the coordination of stomatal regulation under saline conditions, a functional shift that merits further investigation. Conversely, *MELO3C026055* was notably upregulated during fruit ripening, maintaining high expression, indicating a specialized function in fruit development or quality maintenance under saline conditions. These unique expression profiles underscore an adaptation to melon’s fruit-bearing habit and the critical role of its photosynthetic organs.

Light signals affect hormones, proline, and sucrose signaling by regulating the *SOS2*-*PIFs* module or indirectly through pathways such as *COP1*-*HY5*, thereby maximizing the balance between plant salt tolerance and growth [43]. The soybean light signaling factor *GmNF*-*YC14* forms a heterotrimer with *GmNF-YA16* and *GmNF-YB2* to activate the *PYR1*-mediated ABA signaling pathway and enhance salt tolerance [44]. Promoter *cis*-acting element analysis identified a large number of elements related to light, hormones (ABA, SA, JA), and stress (drought, low temperature) responses, with clear differences in element composition among genes. Notably, we identified several Cucurbitaceae-enriched *cis*-elements, such as the fruit-specific C-box and the light-responsive AE-box, which were highly enriched in promoters of fruit-expressed *SOS2* genes (*MELO3C026055*), suggesting a regulatory innovation in melon and related species for coordinating stress responses with reproductive development. The enrichment of ABRE elements in the *MELO3C004428* promoter suggests its potential involvement in ABA-mediated stress response, while the abundance of light-responsive elements in *MELO3C002661 and MELO3C006758* indicates its possible role in growth regulation related to photoperiod or photosynthesis.

These *cis*-element profiles provide a transcriptional basis for the integration of hormone and light signals with SOS2-mediated salt adaptation. For example, the co-occurrence of ABRE and MeJA-responsive elements in promoters of *MELO3C010334* and *MELO3C007208* suggests that these genes may act as hubs cross-talking between ABA and JA pathways-a regulatory feature observed in *Arabidopsis* where SOS2 interacts with ABI2 and is modulated by JA signaling components to fine-tune stress responses [45]. These results explain the functional diversity of *SOS2* genes in responding to various environmental signals at the transcriptional regulation level. It is important to note that the PPI network analysis in this study is limited to a computational prediction. The physical reality of the core interactions, such as those involving *MELO3C010334*, requires further experimental confirmation. Future work should employ techniques like Yeast Two-Hybrid (Y2H) to validate binding affinity and Bimolecular Fluorescence Complementation (BiFC) to observe subcellular localization in *vivo*. Therefore, the current predictive results primarily serve as a hypothetical framework, offering candidate targets and direction for subsequent functional validation.

Through tissue-specific expression analysis and qRT-PCR detection, this study found that melon SOS2 family genes exhibit significant expression patterns under salt and hormone treatments. In vegetative organs, *MELO3C010334* and *MELO3C021231* are highly expressed in roots and hypocotyls, suggesting their potential roles in ion absorption and transport. In contrast, *MELO3C007208* is specifically highly expressed in leaves, possibly related to calcium signaling in chloroplasts. In reproductive organs, *MELO3C006758* is significantly upregulated during early ovary development (0 DAF), and *MELO3C026055* is consistently highly expressed during fruit ripening (29–50 DAF), indicating that this family may also function in floral organ differentiation and fruit quality formation. Salt stress rapidly induced the expression of multiple *SOS2* genes, with *MELO3C010334* showing the most significant response and *MELO3C007208* also sharply upregulated at 12 h, while *MELO3C011108* showed no significant change. This indicates functional specialization among family members in the salt stress response.

In plant abiotic stress responses, SA, ABA, and MeJA play pivotal roles, often interacting with the SOS pathway to orchestrate tolerance [46–48]. Our hormone treatment assays revealed that *MELO3C010334* and *MELO3C007208* respond dynamically to SA, ABA, and MeJA, suggesting their involvement in cross-talk between hormone signaling and ion homeostasis. For instance, the sustained upregulation of *MELO3C010334* under ABA treatment aligns with known ABA-SOS2 interactions in *Arabidopsis*, where ABI2 negatively regulates SOS2 activity [45]. JA and ABA antagonize each other under salt stress, and *ZmEREB57* enhances salt tolerance in maize (*Zea mays* L.) through COI1-dependent JA synthesis [49]. In melon, ABA may similarly modulate CmSOS2 kinase activity to optimize Na^+^/K^+^ balance under salt stress. Meanwhile, the delayed peak response of *MELO3C010334* and *MELO3C007208* to MeJA implies a role in JA-mediated antioxidant or osmotic adjustment processes, which could complement the ion-regulatory function of the SOS pathway, consistent with the conserved yet species-specific regulatory features of *SOS2* reported in *Arabidopsis* and rice [50, 51]. Such multi-hormone responsiveness positions *CmSOS2* genes as integrative nodes in a broader stress-signaling network, coordinating ionic, osmotic, and oxidative stress responses-a mechanism that could be exploited in breeding for protected cultivation, where salinity is often accompanied by other abiotic challenges.

## Conclusions

This study represents the first comprehensive genome-wide identification and functional analysis of the *SOS2* gene family in melon. A total of 20 members were identified, distributed across nine chromosomes, and they exhibited high conservation in phylogeny, gene structure, and conserved domain composition. Promoter analysis revealed a diversity of *cis*-regulatory elements associated with light, hormone, and stress responses, providing a transcriptional basis for their functional divergence in the integration of multiple signaling pathways. Expression profiling demonstrated that *CmSOS2* genes possess distinct tissue-specific and stress-inducible expression patterns. Among them, *MELO3C010334* and *MELO3C007208* showed significant transcriptional responses to treatments with NaCl, ABA, SA, and MeJA. Further functional validation confirmed that overexpression of *MELO3C010334* significantly enhanced salt tolerance in melon, promoted root growth, and improved Na^+^/K^+^ homeostasis in roots. These results systematically elucidate the compositional characteristics and expression patterns of the *SOS2* gene family in melon and reveal the crucial role of *MELO3C010334* in the salt stress response, providing both a key candidate gene and a theoretical foundation for molecular breeding aimed at improving salt tolerance in melon.

## Supplementary Information

Supplementary Material 1.

Supplementary Material 2.

## Data Availability

All data generated or analyzed during this study were included in this published article and the additional files.

## Abbreviations

SOS: Salt Overly Sensitive
ROS: Reactive oxygen species
Mw: Molecular weights
pI: Isoelectric point
Ii: Instability index
GRAVY: Grand average of hydropathicity
Chr: Chromosome
NJ: Neighbor joining
DAF: Days after flowering
qRT-PCR: Quantitative real-time PCR
SA: Salicylic acid
ABA: Abscisic acid
MeJA: Methyl jasmonate
Y2H: Yeast Two-Hybrid
BiFC: Bimolecular Fluorescence Complementation
