# Supplementary material for: Genome-wide analysis of the SOS2 gene family in melon (Cucumis melo L.) and functional characterization of MELO3C010334 in response to salt stress
Source: BMC Plant Biol. 2026 Feb 27;26:613. doi: 10.1186/s12870-026-08456-3 (PMC13049839; doi:10.1186/s12870-026-08456-3)
Supplement: Supplementary file 2 — Supplementary Material 2. [file 12870_2026_8456_MOESM2_ESM.docx]

**Genome-wide analysis of the *SOS2* gene family in melon (*Cucumis melo* L.) and functional characterization of *MELO3C010334* in response to salt stress**

Mengli Yang^1^, Shen Liang^1,*^, Changqing Xuan^1^，Yufan Ma^1^, Kehan Yang^2^, Fei Chen^2^, Man Zhang^3^, Mengyun Hou^3^, Kai Zhao^4,*^

^1^ Institute of Horticultural Research, Henan Academy of Agricultural Sciences, Zhengzhou 450002, China

^2^ College of Life Science and Technology, North Henan Medical University, Xinxiang 453003, China

^3^ College of Horticulture, Henan Agricultural University, Zhengzhou 450046, China

^4^ College of Agronomy, Henan Agricultural University, Zhengzhou 450046, China

^*^Correspondence: Liangshen1@126.com; [zhaok816@1](mailto:zhaokai@henau.edu.cn)63.com

**Supplementary Figures and legends**

**
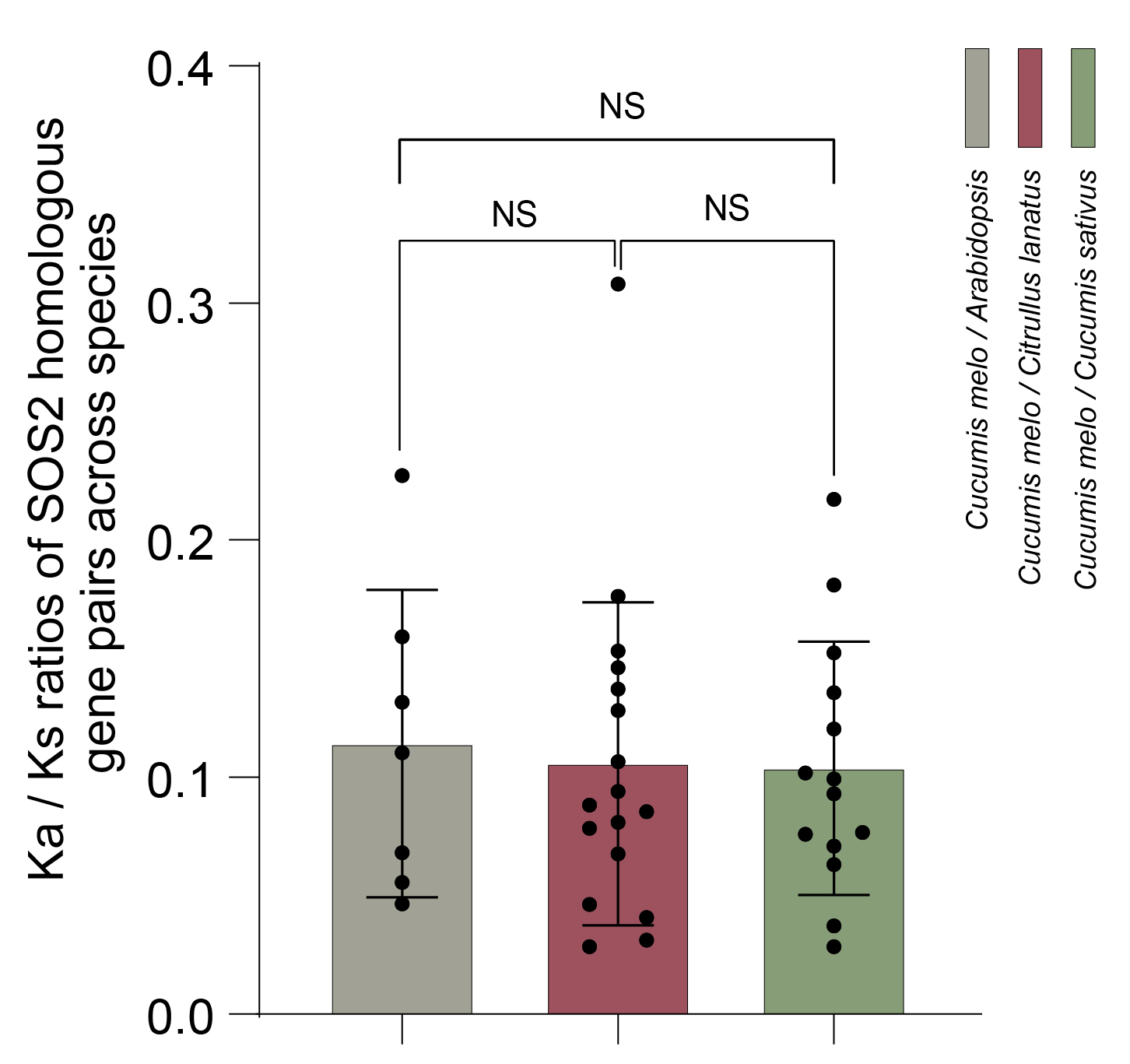
**

**Fig. S1** Ka/Ks ratios of SOS2 homologous gene pairs across species. NS indicates no significant difference.

**
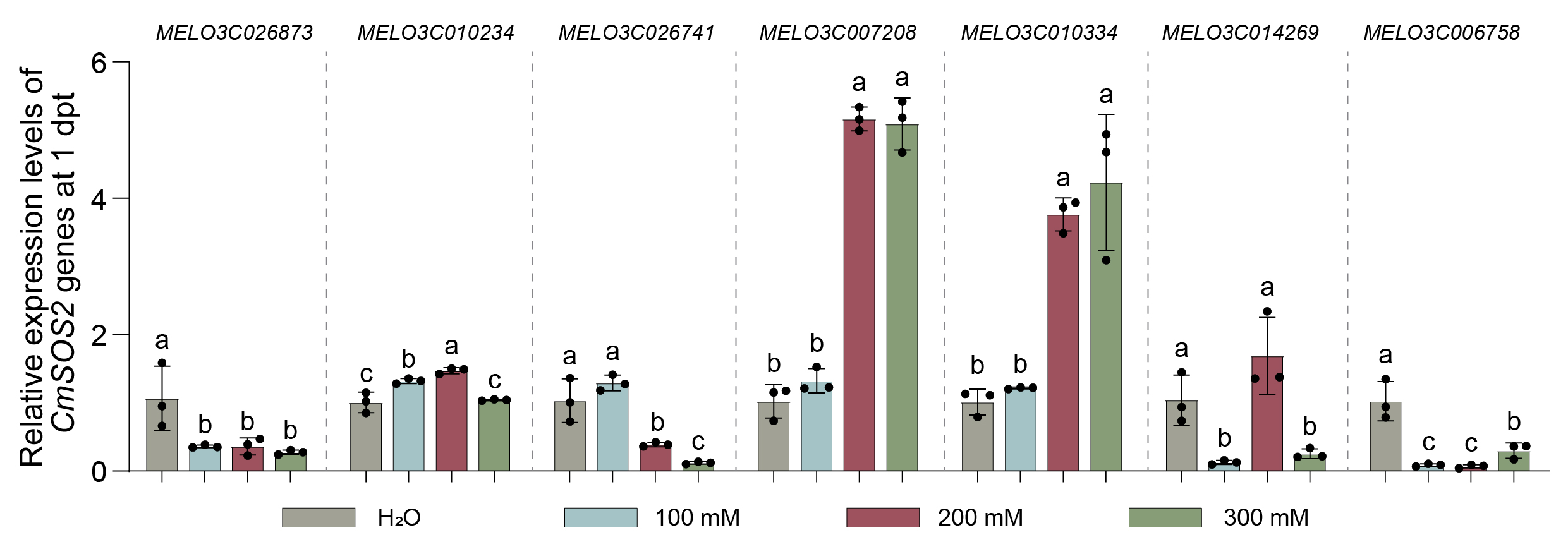
**

**Fig. S2** Relative expression levels of *CmSOS2* genes at 1 day post-treatment under different NaCl concentrations. Data are presented as mean ± SD from three independent biological replicates (n = 3). Different lowercase letters above the bars indicate statistically significant differences among treatments (*p* < 0.05, one-way ANOVA followed by Duncan’s multiple comparison test).


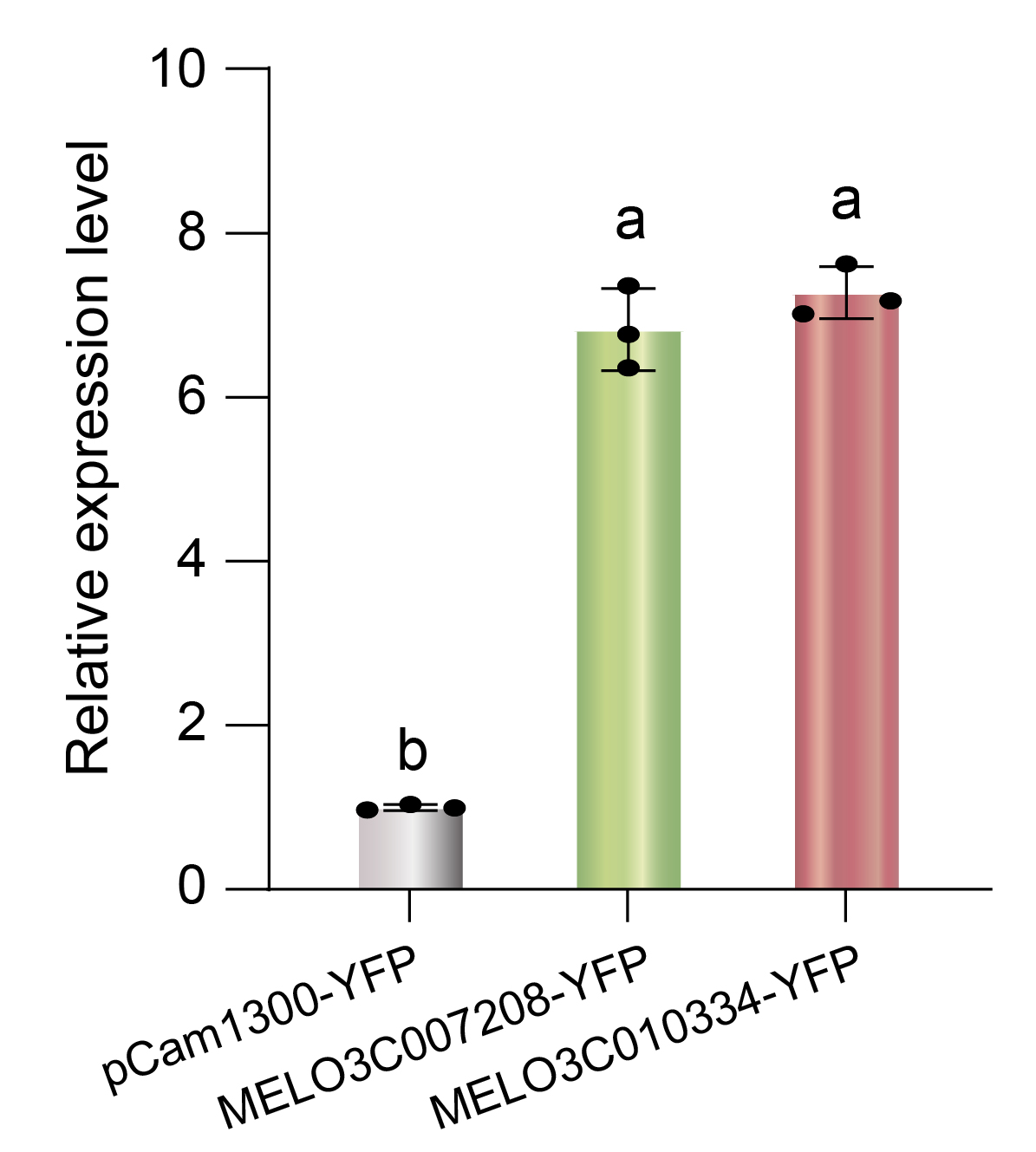


**Fig. S3** Verification of target gene transcript levels in overexpression lines. Relative expression levels of the target gene in root tissues of empty vector control (pCam1300-YFP) and overexpression lines (MELO3C007208-YFP, MELO3C010334-YFP) were determined by qRT‑PCR. Bars represent means, and error bars indicate ± SD of three biological replicates. Different letters above bars denote significant differences among groups (*P* < 0.05, one-way ANOVA followed by Duncan’s multiple comparison test).
